# Machine learning for functional outcome prediction after vestibular schwannoma surgery: a systematic review and diagnostic test accuracy meta-analysis

**DOI:** 10.1007/s11060-026-05747-5

**Published:** 2026-08-24

**Authors:** Shiva A. Nischal, Shaan Patel, Musa China, Kush M. Kale, Yi Hein Chai, Santosh Guru, William R. Muirhead, Patrick Grover

**Affiliations:** 1 Department of Physiology, Anatomy & Genetics, Medical Sciences Division, University of Oxford, Oxford, UK; 2 Department of Neurological Surgery, Thomas Jefferson University Hospital, Philadelphia, PA USA; 3 Victor Horsley Department of Neurosurgery, National Hospital for Neurology and Neurosurgery, London, UK; 4 University College London Medical School, University College London, London, UK; 5 School of Clinical Medicine, University of Cambridge, Cambridge, UK

**Keywords:** Vestibular schwannoma, Machine learning, Deep learning, Neural network, Hearing preservation, Facial nerve palsy

## Abstract

**Purpose:**

Machine learning (ML) models have been increasingly applied to predict postoperative facial nerve dysfunction and hearing preservation after vestibular schwannoma (VS) surgery. However, reported performance varies substantially, and the overall diagnostic accuracy and clinical reliability of these models remain uncertain. We conducted a systematic review and diagnostic test accuracy meta-analysis to characterise the current state and methodological readiness of ML-based prediction of these outcomes.

**Methods:**

PubMed, Embase, and CENTRAL were searched from inception to February 2026. Studies evaluating ML-based prediction of facial nerve function or hearing preservation following VS surgery were included. Diagnostic performance metrics were pooled using random-effects generalised linear mixed models. Sensitivity, specificity, diagnostic odds ratio, and AUC were synthesised, and SROC curves were constructed. The prespecified primary synthesis pooled the single best model per study; small-study effects were assessed with Deeks’ test. Risk of bias (PROBAST) and certainty of evidence (GRADE) were assessed.

**Results:**

Ten retrospective cohort studies encompassing 1270 patients and 56 ML models met inclusion criteria. In the prespecified primary analysis pooling the single best model per study, the summary AUC was 0.91 for facial nerve dysfunction (sensitivity 0.89, specificity 0.86) and 0.92 for hearing preservation (sensitivity 0.88, specificity 0.96). Pooling all models on held-out test data gave a facial nerve AUC of 0.81; test-set data were too sparse for a stable hearing estimate, for which only training performance could be pooled (AUC 0.79). Tumour size, age, tumour location, and baseline hearing status were the most frequently identified influential predictors. Most studies were at unclear or high risk of bias (PROBAST has no intermediate “moderate” category), and certainty of evidence was moderate for facial nerve dysfunction and low for hearing preservation, the latter reflecting significant small-study effects (Deeks’ *p* = 0.004).

**Conclusion:**

ML-based models demonstrate promising discrimination for predicting postoperative facial nerve and hearing outcomes after VS surgery. However, heterogeneity, limited external validation, and inconsistent reporting of calibration constrain inference regarding transportability and clinical implementation.

**Supplementary Information:**

The online version contains supplementary material available at https://doi.org/10.1007/s11060-026-05747-5.

## Introduction

Vestibular schwannomas (VSs) are benign neoplasms arising from the vestibular division of the eighth cranial nerve and represent the most common tumour of the cerebellopontine angle [1]. Widespread magnetic resonance imaging (MRI) has led to increased incidental detection, with population-level prevalence estimates now exceeding 1 in 500 individuals [2]. Although histologically benign, VSs are clinically consequential due to their intimate anatomical relationship with the facial and cochlear nerves, rendering facial nerve dysfunction and hearing loss the principal determinants of postoperative quality of life [3].

Over recent decades, management philosophy has shifted from maximal tumour removal toward functional preservation [4, 5]. Contemporary management strategies, including observation, stereotactic radiosurgery, and microsurgical resection, are individualised according to tumour characteristics, symptom burden, patient preference, and anticipated functional risk [3, 6, 7]. For patients undergoing surgery, preservation of facial nerve integrity and serviceable hearing remains a central objective. Despite advances in skull base technique, intraoperative neurophysiological monitoring (IONM), and surgical approach selection, clinically meaningful rates of postoperative facial nerve dysfunction and hearing deterioration persist [8, 9].

Traditional prognostic efforts have relied on univariate associations and multivariable regression models incorporating tumour size, age, surgical approach, and baseline functional status [10–15]. While informative at a cohort level, these approaches demonstrate only modest discrimination when applied to individual patients. Machine learning (ML) methods have emerged as an alternative framework capable of modeling nonlinear relationships and high-dimensional feature interactions [16]. In the context of VS surgery, ML models have been developed to predict postoperative facial nerve outcomes and hearing preservation using combinations of clinical, radiographic, and operative variables [17]. However, reported performance varies substantially across studies, and external validation remains inconsistent.

Accordingly, a rigorous synthesis of the available evidence is required to clarify the diagnostic performance of ML-based models for functional outcome prediction after VS surgery. In this PRISMA-DTA-compliant systematic review and meta-analysis, we aimed to characterise the discrimination reported for these models while accounting for within-cohort non-independence, map the heterogeneity that conditions whether the estimates can be pooled, and appraise methodological quality, risk of bias, and certainty of evidence, establishing the field’s readiness for synthesis rather than delivering a single deployable estimate. By situating ML-based prediction within the broader framework of functional preservation in skull base oncology, this study characterises the current evidentiary position of data-driven prognostication in VS surgery and its readiness to inform patient care.

## Materials & methods

### Study design and reporting standards

This systematic review and diagnostic test accuracy meta-analysis was conducted in accordance with PRISMA-DTA guidelines [18] and appraised using the AMSTAR-2 framework [19]. The protocol was prospectively registered with PROSPERO (CRD420261293466).

### Search strategy

PubMed, Embase, and CENTRAL were searched from database inception to 1st February 2026. Search strategies combined controlled vocabulary (MeSH/Emtree terms) with free-text keywords relating to artificial intelligence, ML, deep learning (DL), neural networks (NNs), automation, VS, facial nerve, and hearing loss. Search strategies were adapted for each database (Supplementary Table 1). Searches were restricted to human studies published in English. Reference lists of included studies and relevant prior reviews were manually screened to identify additional eligible studies.

### Eligibility criteria

Studies were included if they (i) included patients undergoing VS surgery, (ii) employed ML methods to predict postoperative facial nerve function and/or hearing preservation, and (iii) reported extractable diagnostic performance metrics (including sensitivity, specificity, area under the curve (AUC), accuracy, or confusion-matrix elements).

Studies were excluded if they did not utilise ML-based prediction methods, did not report facial nerve or hearing outcomes separately, did not provide sufficient data for diagnostic accuracy synthesis, included fewer than 10 patients, or were case reports, conference abstracts, reviews, editorials, or non-English publications.

### Study selection

Two reviewers independently screened titles and abstracts using Rayyan [20], followed by full-text assessment. Disagreements were resolved by consensus with a third reviewer. Inter-rater reliability was quantified using Cohen’s kappa (κ = 0.89).

### Data extraction

Data was extracted independently using a standardised template. Extracted variables included: study characteristics (author, year, country, study design), cohort characteristics (sample size, age, sex, tumour size, preoperative House-Brackmann grade, preoperative hearing metrics (pure tone average, word recognition score, American Academy of Otolaryngology-Head and Neck Surgery (AAO-HNS) classification), surgical approach, extent of resection), model characteristics (algorithm type, feature domains (clinical, radiographic, radiomic), training and test splits, cross-validation strategy, feature selection methods), and performance metrics (true positive, false positive, true negative, false negative, sensitivity, specificity, AUC, accuracy). When multiple models were reported within a study, each model was extracted separately. Models reported without extractable 2 × 2 data (for example, the conventional classifiers alongside the sequence models in Song et al.) were not extracted. No two included cohorts shared patients. Training and testing datasets were analysed independently to distinguish internal optimisation from held-out validation performance. We distinguish apparent (training), internal cross-validation, held-out internal, and external validation; no included study reported external validation, and performance derived from internal cross-validation or a single held-out split. Feature importance was synthesised descriptively by extracting the top-ranked predictors each study’s best performing model – given anticipated heterogeneity in attribution methods, quantitative pooling of feature weights was not undertaken.

### Statistical analysis

Diagnostic performance was synthesised using random-effects generalised linear mixed models (GLMMs), pooling sensitivity and specificity with 95% confidence intervals (CIs). From confusion-matrix data, pooled positive likelihood ratios (PLRs), negative likelihood ratio (NLRs), and diagnostic odds ratios (dORs) were calculated. Summary receiver operating characteristic (SROC) curves were generated, and AUC was reported as a global measure of discrimination. Training and test datasets were analysed separately. Given anticipated heterogeneity in outcome definitions and follow-up intervals, threshold-robust measures (SROC, AUC) were prioritised for interpretation. Statistical significance was defined as α < 0.05. Because pooling several models per cohort violates the independence assumption and narrows the confidence intervals, we prespecified as the primary analysis a synthesis of the single highest-performing model per study (by reported AUC), retaining the all-models synthesis as a precision-inflating sensitivity analysis. Because few studies reported both, the training and test syntheses comprise largely different studies and are not paired. All analyses were performed using R version 4.3.2 [21].

### Risk of bias and certainty of evidence

Risk of bias and applicability were assessed using PROBAST [22] across four domains: participants, predictors, outcome, and analysis. Two reviewers independently performed assessments, with disagreements resolved by consensus. Certainty of evidence for each pooled diagnostic outcome was evaluated using GRADE (adapted for diagnostic test accuracy studies) [23]. Small-study effects were evaluated using Deeks’ funnel plot asymmetry test.

### Assessment of clinical utility

Clinical utility was explored using Fagan nomograms to estimate post-test probabilities based on pooled likelihood ratios and an assumed pre-test probability of 50.0%. This value is a neutral reference point rather than the empirical event prevalence, which varied across cohorts and outcome definitions (approximately 10–35% for facial nerve dysfunction and 30–55% for hearing non-preservation); post-test probabilities should therefore be interpreted across this plausible range rather than at a single anchor.

## Results

### Study selection

**Fig. 1:**
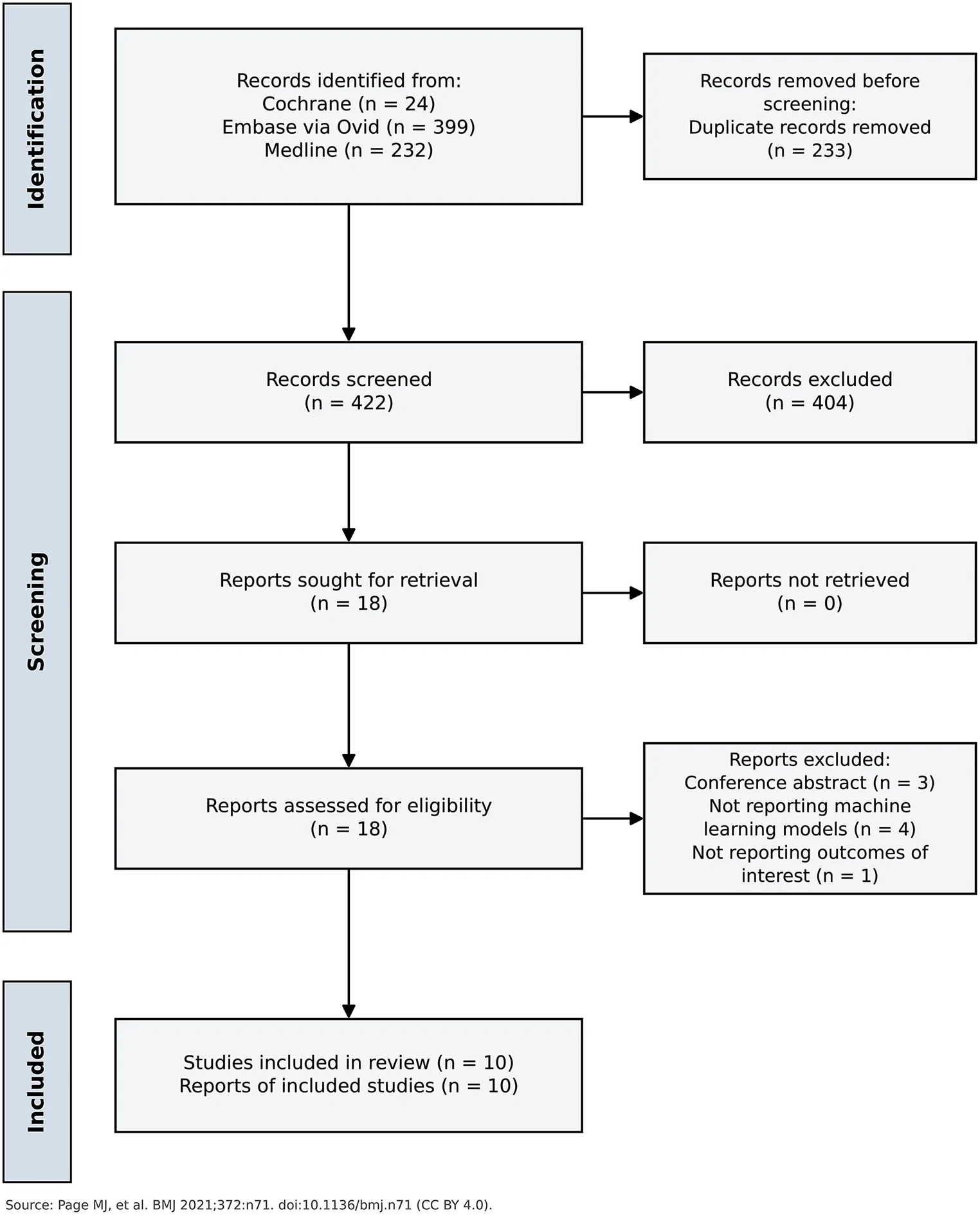
PRISMA flow diagram of study selection. Preferred reporting items for systematic reviews and meta-analyses (PRISMA) 2020 flow diagram illustrating the identification, screening, eligibility assessment, and final inclusion of studies evaluating machine learning models for prediction of postoperative facial nerve dysfunction and hearing preservation following vestibular schwannoma surgery. Reasons for exclusion at the full-text stage are specified

A total of 655 records were identified through database searching. After removal of duplicates and screening, 10 retrospective cohort studies [17, 24–32] met inclusion criteria (Fig. 1). No additional studies were identified through manual reference screening. Across included studies, 1270 patients were analysed. The 56 unique models reported correspond to 65 model–dataset evaluations (48 facial, 17 hearing), as several were evaluated on both training and test data; counts below are at the model–dataset level unless stated.

### Study and cohort characteristics

Baseline characteristics are summarised in Table 1.

**Table 1:** Baseline study and cohort characteristics. Summary of study design, demographic characteristics, tumour features, operative variables, and pre- and postoperative functional outcomes across included cohorts evaluating machine learning models prediction of postoperative facial nerve dysfunction following vestibular schwannoma surgery. Continuous variables are reported as mean ± standard deviation or mean (range), as provided in the original studies. Categorical variables are presented as counts

Study	Country	Predicted outcome	Study design	Population size (n)	Age (years)	Sex (M/F)	Tumour diameter (mm)	Surgical approach (n)	Gross-total resection (n)	Postoperative HB grade, short-term	Preoperative PTA (dB)	Preoperative WRS (%)
Song (2025)	China	Facial nerve	RC	117	47.0 (35.0–55.0)	43/74	NR	RS 117, TL 0, MF 0	97	I-II 90/III-VI 26	NR	NR
Przepiorka (2025)	Poland	Facial nerve	RC	256	49.0 (38.0–58.0)	98/158	NR	RS 253, TL 4, MF 0	241	27/40/51/81/29/28	NR	NR
Heman-Ackah (2024)	USA	Facial nerve	RC	242	53.6 ± 14.8	112/130	NR	RS 168, TL 74, MF 0	NR	NR	NR	NR
Zohdy (2024)	USA	Facial nerve	RC	71	43.1 ± 12.1	31/40	NR	RS 49, TL 12, MF 10	12	24/17/12/9/6/3	NR	NR
Wang M-Y (2023)	China	Facial nerve	RC	110	50.9 ± 10.2	42/68	30.3 ± 9.5	RS 110, TL 0, MF 0	105	I-II 83/III-IV 110	NR	NR
Wang J (2022)	China	Facial nerve	RC	128	NR	NR	NR	NR	NR	NR	NR	NR
Yang (2023)	China	Hearing preservation	RC	79	NR	NR	NR	NR	NR	NR	NR	NR
Dixon (2022)	USA	Hearing preservation	RC	144	47.7 ± 10.5	93/51	9.8 ± 4.5	RS 0, TL 0, MF 144	NR	NR	23.1 ± 18.7	94.6 ± 9.0
Cha (2020)	South Korea	Hearing preservation	RC	50	47.2 ± 11.5	19/31	13.1 ± 6.2	RS 23, TL 0, MF 27	NR	NR	26.6 ± 15.6	81.0 ± 22.9
Lim (2024)	South Korea	Hearing preservation	RC	73	53.5 ± 12.9	35/38	15.5 ± 11.9	NR	NR	NR	54.1 ± 27.6	62.9 ± 35.8

Abbreviations: RC, retrospective cohort; M, male; F, female; HB, House-Brackmann; PTA, pure tone average; WRS, word recognition score; RS, retrosigmoid; TL, translabyrinthine; MF, middle fossa; NR, not reported

All studies were retrospective cohort designs. Four studies originated from China, three from the United States, two from South Korea, and one from Poland. Mean patient age across studies was 49.0 years, and 55.5% were female. Operative approach varied by study, though retrosigmoid approaches were most frequently reported (72.7%, 720/991), followed by middle fossa (18.3%, 181/991) and translabyrinthine (9.1%, 90/991) approaches. Gross total resection was achieved in 35.8% (455/1270) of cases.

Facial nerve function was uniformly assessed using the House-Brackmann grading system and was most commonly grades I (82.9%, 271/327) or II (14.4%, 47/327) preoperatively. Hearing outcomes were evaluated using pure tone average (preoperative mean 34.6 dB), word recognition score (preoperative mean 79.5%), or AAO-HNS classification.

### Overview of machine learning models

Across the 10 included studies, 56 unique models yielded 65 model-dataset evaluations. Of these, facial nerve prediction encompassed 48 evaluations from 6 studies [24, 26–28, 31, 32], and hearing preservation 17 models from 4 studies [17, 25, 29, 30]. Model characteristics are summarised in Table 2 and Supplementary Table 2.

**Table 2:** Model development characteristics, feature domains, and key predictors across included studies

Study	Predicted outcome	Models	Class-imbalance handling	Cross-validation strategy	Input parameters	Reference standard	Top predictors (descending order)
Song (2025)	Facial nerve	SVM; LR; DT; naïve Bayes; bagging; 1D-CNN; 1D-CNN-LSTM; 1D-CNN-GRU	SMOTE-ENN	Train/test split (80/20)	Radiomic shape features (major axis length, mesh volume, voxel volume); first-order intensity features (energy, total energy); texture features (GLRLM, GLSZM, GLDM)	HB grade	1) GLDM non-uniformity 2) GLDM entropy 3) GLDM grey-level non-uniformity 4) GLSZM size-zone non-uniformity 5) GLSZM zone entropy
Przepiorka (2025)	Facial nerve	XGBoost	Class weighting (XGBoost scale_pos_weight parameter)	5-fold cross-validation	Clinical and operative variables: age; sex; presenting symptoms (facial nerve dysfunction, tinnitus, dizziness); tumour size, volume, laterality; surgical approach and position; complications; preoperative HB grade; Samii hearing scale; ABR	HB grade	1) Short-term facial nerve function 2) Intraoperative difficulty 3) Incidental presentation 4) Age at surgery 5) Tumour volume
Heman-Ackah (2024)	Facial nerve	RF	SMOTE	Train/test split (90/10)	Demographic and operative variables: age; sex; BMI; tumour size and linear measurements; surgical approach; case duration; residual tumour	HB grade	1) BMI 2) Case duration 3) Tumour growth towards brainstem 4) Age
Zohdy (2024)	Facial nerve	ANN; RF; LR	NR	Stratified train/test split (75/25) with 10-fold cross-validation	Electrophysiological and clinical variables: P/D stimulation ratio; age; sex; tumour size; preoperative HB grade	HB grade	1) Mentalis P/D ratio 2) Orbicularis oris P/D ratio 3) Nasalis P/D ratio 4) Frontalis P/D ratio
Wang M-Y (2023)	Facial nerve	CNN; LightGBM; SVM; SGD; KNN; DT; RF; ET; LR	NR	Train/test split (70/30) with 10-fold cross validation	Age; surgical strategy; tumour diameter; radiomic features	HB grade	NR
Wang J (2022)	Facial nerve	LR; SVM; CART; RF; XGBoost	SMOTE-ENN	5-fold cross-validation	Clinical and radiological variables: age; sex; tumour size; tumour consistency (solid versus cystic); brainstem compression; internal auditory canal morphology; TFIAC grading; Samii grading; cerebellar and posterior cranial nerve symptoms; preoperative neurological grade	HB grade	1) Tumour size 2) TFIAC grade 3) Age
Yang (2023)	Hearing preservation	DNN; CART; XGBoost	NR	Train/test split (70/30) with 5-fold cross validation	Demographic and tumour-related variables: age; hypertension, diabetes; tumour size; tumour consistency (solid versus cystic); tumour morphology; Samii grade; TFIAC grade; preoperative hearing class; presenting symptoms; medication use; surgical position	Binary	1) Tumour size 2) Age 3) Preoperative AAO-HNS hearing class 4) Tumour cystic status 5) Number of preoperative symptoms
Dixon (2022)	Hearing preservation	RF; CART	NR	Train/test split (80/20)	Clinical and audiometric variables: age, sex, BMI; preoperative symptoms (hearing loss, tinnitus, dizziness); PTA; WRS; tumour laterality; Koos grade	PTA & WRS	1) Preoperative PTA 2) Preoperative WRS 3) Preoperative AAO-HNS hearing class 4) History of migraine 5) Tumour proportion anterior to internal auditory canal mid-pole
Cha (2020)	Hearing preservation	DNN; GBM; RF; SVM; LR; GLM; DT	NR	5-fold cross validation	Audiometric and vestibular variables: PTA threshold; WRS; SDT; MCL; ABR; VEMPs; CP; maximum tumour diameter; surgical approach	PTA & WRS	1) Preoperative WRS 2) VEMP 3) Tumour size 4) CP 5) I-V interval
Lim (2024)	Hearing preservation	RF	NR	Train/test split (70/30)	Radiomic features: histogram-based; shape-based; texture-based; filter-derived metrics	AAO-HNS	1) Log skewness (radiomic feature) 2) Maximum 3D diameter 3) Skewness slope

Summary of machine learning (ML) models developed to predict postoperative facial nerve dysfunction or hearing preservation following vestibular schwannoma surgery. For each study, the predicted outcome, algorithm(s), class imbalance strategy, input feature domains, reference standard, and top-ranked predictors (descending order of importance in the best-performing model) are reported. Where applicable, radiomic feature classes (shape, first-order intensity, texture) and electrophysiological variables are specified. “NR” denotes not reported

Abbreviations: ABR, auditory brainstem response; AAO-HNS, American Academy of Otolaryngology-Head and Neck Surgery; ANN, artificial neural network; BMI, body mass index; CART, classification and regression tree; CNN, convolutional neural network; CP, canal paresis; DNN, deep neural network; DT, decision tree; ET, extra trees; GBM, gradient boosting machine; GLDM, grey level dependence matrix; GLM, generalised linear model; GLRLM, grey level run length matrix; GLSZM, grey level size zone matrix; GRU, gated recurrent unit; HB, House-Brackmann; I-V interval, interpeak latency between waves I and V; KNN, k-nearest neighbour; LightGBM, light gradient boosting machine; LR, logistic regression; LSTM, long short-term memory; MCL, most comfortable level; PTA, pure tone average; P/D ratio, proximal-to-distal stimulation ratio; RF, random forest; SDT, speech detection threshold; SGD, stochastic gradient descent; SMOTE, synthetic minority oversampling technique; SMOTE-ENN, synthetic minority oversampling with edited nearest neighbours; SVM, support vector machine; TFIAC, tumour filling of the internal auditory canal grading; VEMP, vestibular-evoked myogenic potential; WRS, word recognition score; XGBoost, extreme gradient boosting

For facial nerve outcomes, 14 models were evaluated using training datasets and 34 models were assessed on independent test datasets. For hearing preservation, 14 models were evaluated using training data and 3 models were assessed on independent test datasets. Algorithms spanned a range of supervised learning approaches, including random forest (RF), support vector machine (SVM), logistic regression (LR), gradient boosting, decision trees, artificial NNs, convolutional NNs, and hybrid DL architectures. Several studies implemented radiomics-based pipelines, frequently employing PyRadiomics for structured feature extraction from MRI datasets.

Among the 14 facial nerve training-set models, EM was most frequent (28.6%, 4/14), followed by regression models (21.4%, 3/14), SVM (14.3%, 2/14), DT (14.3%, 2/14), KNN (14.3%, 2/14) and DL (7.1%, 1/14). AUC and ACC ranged from 0.72–0.95 and 0.59–0.87, respectively. Sensitivity and specificity varied from 0.59–0.91 and 0.50–0.86, respectively.

Among the 34 facial nerve test-set models, DL was most frequent (32.4%, 11/34), followed by EM (23.5%, 8/34), then regression models (11.8%, 4/34), DT (11.8%, 4/34), SVM (11.8%, 4/34), NB (5.9%, 2/34) and KNN (2.9%, 1/34). The AUC and ACC ranged from 0.64–0.95 and 0.63–1.00, respectively. Sensitivity and specificity varied from 0.41–1.00 and 0.65–1.00, respectively.

Among the 17 hearing models, EM was most frequent (35.3%, 6/17), followed by DT (23.5%, 4/17), DL (17.6%, 3/17), regression models (17.6%, 3/17), and SVM (5.9%, 1/17). The AUC was 0.66 (from one study) and ACC ranged from 0.36–0.94. Sensitivity and specificity ranged from 0.26–0.93 and 0.23–1.00, respectively.

### Pooled diagnostic performance

Training-set (apparent) performance, reported below for completeness, overestimates true performance and is not advanced as a generalisable estimate.

**Fig. 2:**
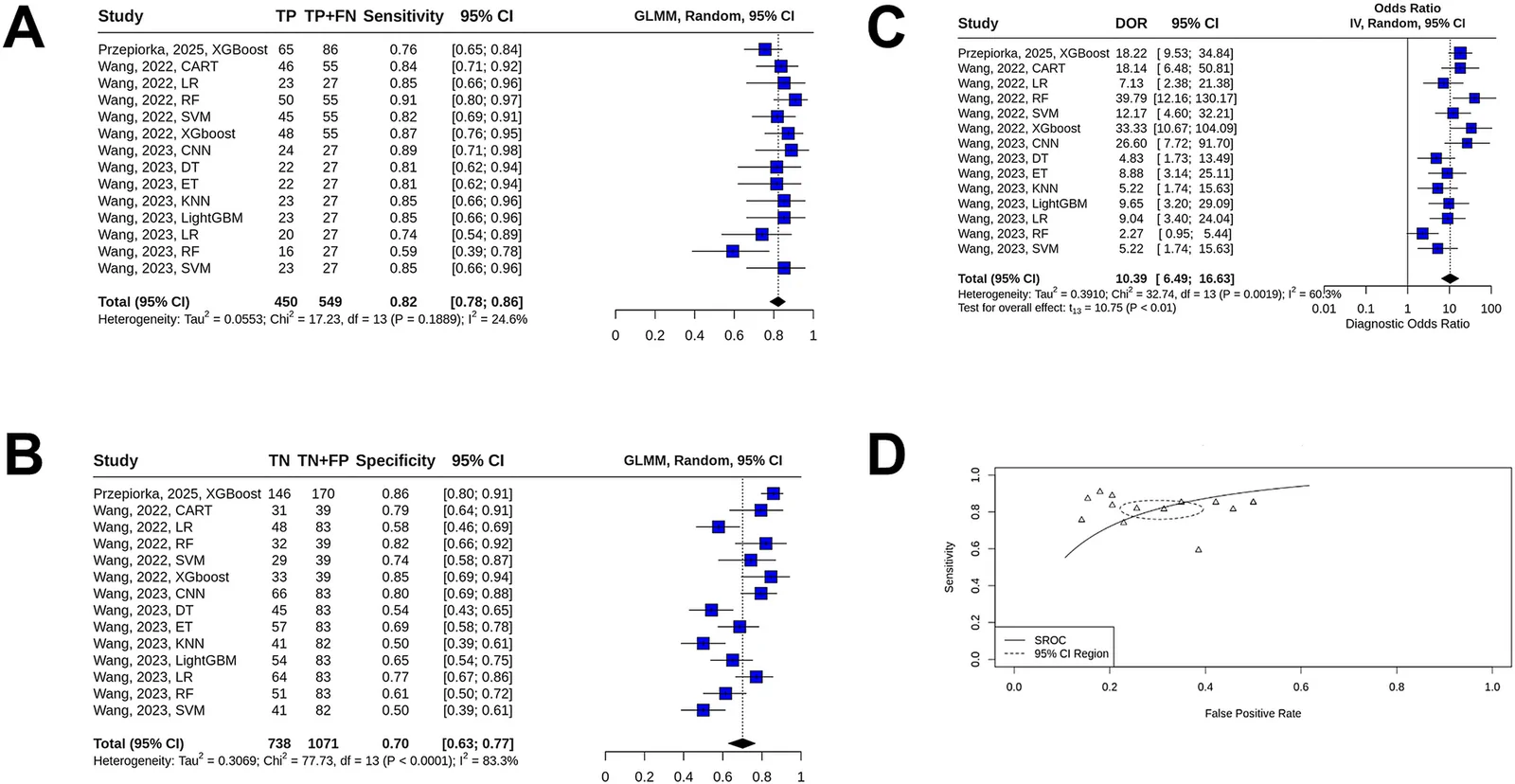
Pooled predictive performance for facial nerve dysfunction (training datasets). Forest plots derived from random-effects generalised linear mixed models (GLMMs) summarising (**A**) sensitivity, (**B**) specificity, and (**C**) diagnostic odds ratio (dOR) for machine learning-based prediction of postoperative facial nerve dysfunction using training datasets. Individual study estimates are shown as squares proportional to study weight. Horizontal lines indicate 95% confidence intervals. Diamond represents pooled estimate for overall effect. (**D**) Summary receiver operating characteristic (SROC) curve depicting the overall predictive performance for facial nerve dysfunction, with the summary operating point (pooled sensitivity and specificity), 95% confidence region, and the area under the curve (AUC). Abbreviations: IV, inverse variance; GLMM, generalised linear mixed model

Three studies [26–28] comprising 14 models contributed to pooled analysis of training performance for facial nerve dysfunction. The pooled sensitivity was 0.82 (95% CI: 0.78–0.86; I^2^ = 24.6%; *p* < 0.0001) and specificity was 0.70 (95% CI: 0.63–0.77; I^2^ = 83.3%; *p* < 0.0001), corresponding to a dOR of 10.39 (95% CI: 6.49–16.63; I^2^ = 60.3%; *p* < 0.01) and AUC of 0.75 (95% CI: 0.73–0.78) (Fig. 2).

**Fig. 3:**
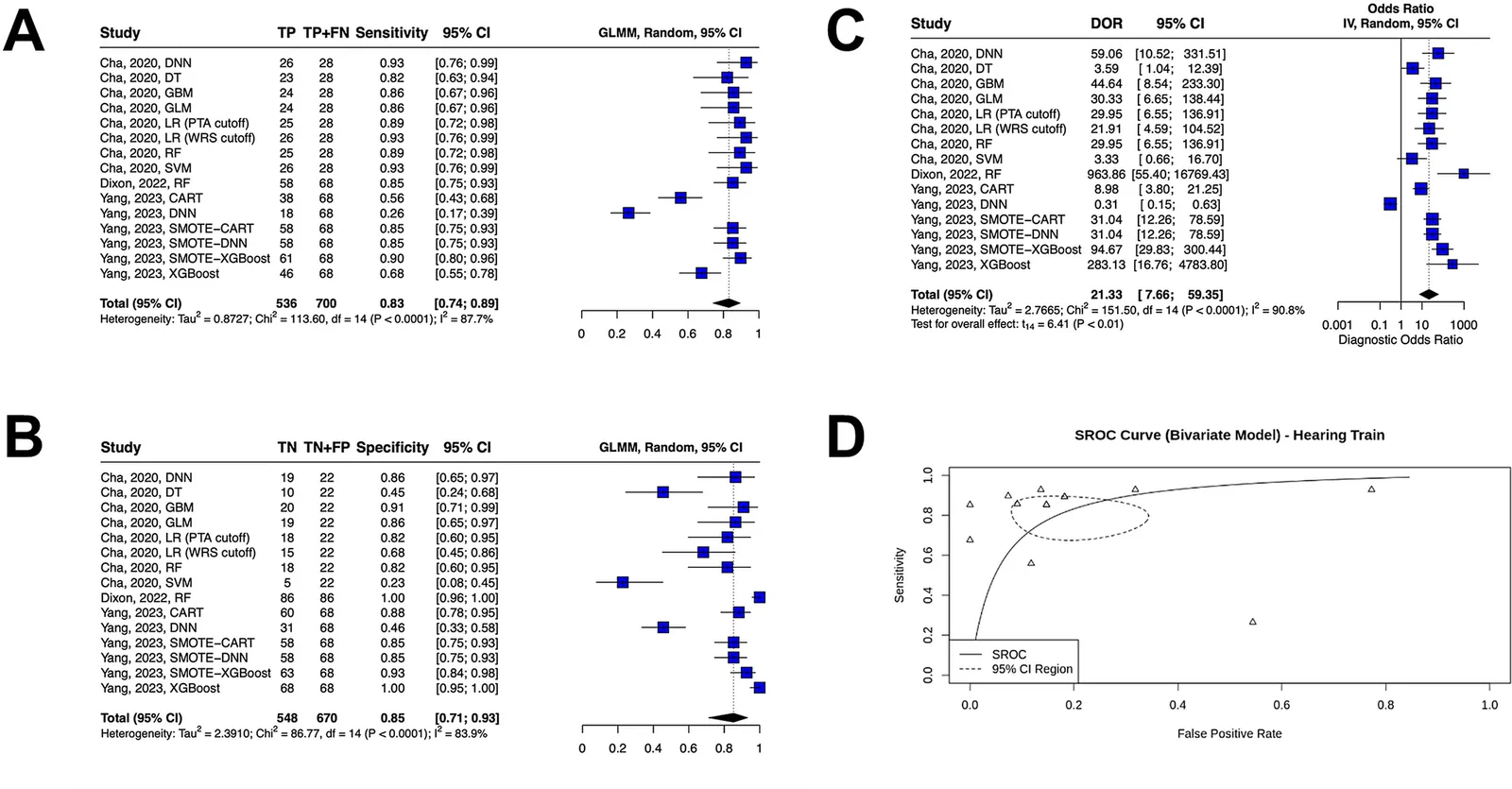
Pooled predictive performance for hearing preservation (training datasets). Forest plots derived from random-effects generalised linear mixed models (GLMMs) summarising (**A**) sensitivity, (**B**) specificity, and (**C**) diagnostic odds ratio (dOR) for machine learning-based prediction of postoperative hearing preservation using training datasets. Individual study estimates are shown as squares proportional to study weight. Horizontal lines indicate 95% confidence intervals. Diamond represents pooled estimate for overall effect. (**D**) Summary receiver operating characteristic (SROC) curve depicting the overall predictive performance for hearing preservation, with the summary operating point (pooled sensitivity and specificity), 95% confidence region, and the area under the curve (AUC). Abbreviations: IV, inverse variance; GLMM, generalised linear mixed model

Three studies [17, 25, 30] contributing 15 models were included in pooled analysis of hearing preservation performance (training data). The pooled sensitivity was 0.83 (95% CI: 0.74–0.89; I^2^ = 87.7%; *p* < 0.0001) and specificity was 0.85 (95% CI: 0.71–0.93; I^2^ = 83.9%; *p* < 0.0001), yielding a pooled dOR of 21.33 (95% CI: 7.66–59.35; I^2^ = 90.8%; *p* < 0.0001) and AUC of 0.79 (95% CI: 0.77–0.81) (Fig. 3).

**Fig. 4:**
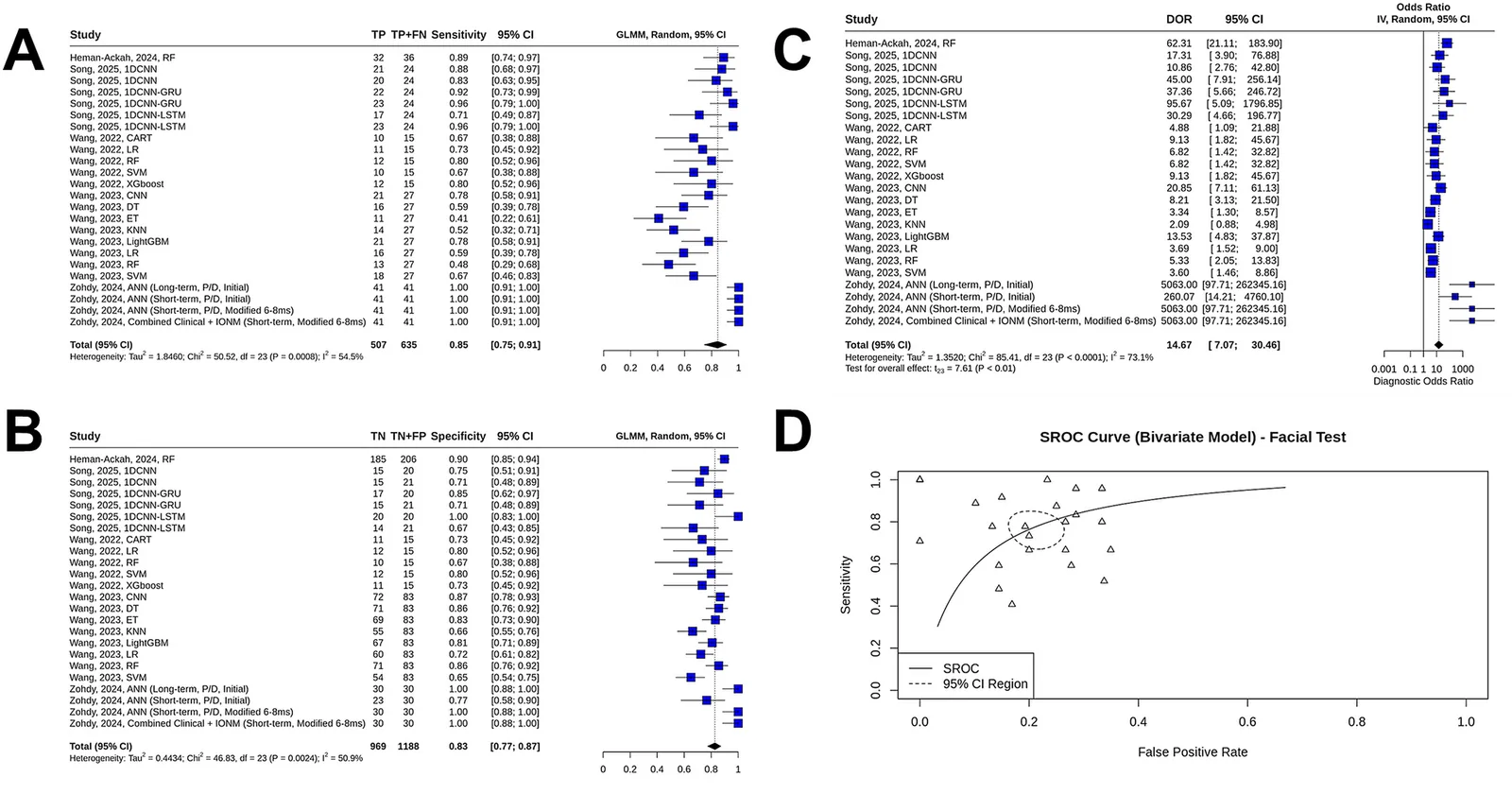
Pooled predictive performance for facial nerve dysfunction (test datasets). Forest plots derived from random-effects generalised linear mixed models (GLMMs) summarising (**A**) sensitivity, (**B**) specificity, and (**C**) diagnostic odds ratio (dOR) for machine learning-based prediction of postoperative facial nerve dysfunction using independent test datasets. Individual study estimates are shown as squares proportional to study weight. Horizontal lines indicate 95% confidence intervals. Diamond represents pooled estimate for overall effect. (**D**) Summary receiver operating characteristic (SROC) curve showing the overall predictive performance for FND, with the summary operating point (pooled sensitivity and specificity), 95% confidence region, and the area under the curve (AUC). Abbreviations: IV, inverse variance; GLMM, generalised linear mixed model

Five studies [24, 26, 27, 31, 32] comprising 24 models were included in pooled analysis of test-set performance for facial nerve dysfunction. The pooled sensitivity was 0.85 (95% CI: 0.75–0.91; I^2^ = 54.5%; *p* < 0.001) and specificity was 0.83 (95% CI: 0.77–0.87; I^2^ = 50.9%; *p* < 0.01), with a dOR of 14.67 (95% CI: 7.07–30.46; I^2^ = 73.1%; *p* < 0.0001) and an AUC of 0.81 (95% CI: 0.79–0.83) (Fig. 4).

### Best-performing model analysis

This best-model synthesis was the prespecified primary analysis: pooling one estimate per cohort respects between-study independence, whereas the all-models estimates above inflate precision.

For hearing preservation, pooled sensitivity was 0.88 (95% CI 0.83–0.92, I^2^ = 0.0%) and specificity was 0.96 (95% CI 0.80–0.99, I^2^ = 0.0%), with a dOR of 129.97 (95% CI 50.05–337.50; I^2^ = 8.7%), accuracy of 0.92 (95% CI 0.89–0.94, I^2^ = 0.0%), and a summary AUC of 0.92 (Supplementary Fig. 1).

For facial nerve dysfunction, pooled sensitivity was 0.89 (95% CI 0.81–0.94, I^2^ = 30.0%) and specificity was 0.86 (95% CI 0.81–0.89, I^2^ = 33.8%), with a dOR of 31.53 (95% CI 18.80–52.87; I^2^ = 45.8%), accuracy of 0.87 (95% CI 0.82–0.91, I^2^ = 14.5%), and AUC of 0.91 (95% CI 0.89–0.98) (Supplementary Fig. 2).

### Feature importance patterns

Feature ranking analyses demonstrated convergence across studies. For facial nerve outcomes, age (3/5 studies), tumour size (2/5 studies), and tumour location (2/5 studies) were the most consistently identified predictors. For hearing preservation, tumour size (3/4 studies) and preoperative hearing metrics (2/4 studies) were most frequently prioritised (Table 2).

### Clinical utility

Clinical applicability was evaluated using Fagan nomograms based on pooled likelihood ratios and an assumed pre-test probability of 50.0% (a neutral reference, not the empirical prevalence). Under this assumption, a positive ML prediction increased the post-test probability to 81.3% for facial nerve dysfunction and 92.4% for hearing outcomes. Conversely, a negative prediction reduced post-test probabilities to 12.3% and 10.0%, respectively (Supplementary Fig. 3).

### Risk of bias and certainty of evidence

Overall risk of bias (PROBAST) was low in one study, high in one, and unclear in 8; PROBAST defines no intermediate “moderate” category (Supplementary Table 3). Deeks’ funnel plot asymmetry analysis demonstrated possible publication bias for hearing outcomes (*p* = 0.004) and borderline asymmetry for facial nerve outcomes (*p* = 0.05) (Supplementary Fig. 4). The significant hearing asymmetry indicates small-study effects and probable publication bias, and is the main reason the pooled hearing estimate is not advanced as reliable. Certainty of evidence (GRADE) was moderate for facial nerve dysfunction and low for hearing preservation, downgraded for risk of bias and, for hearing, small-study effects (Supplementary Table 4).

## Discussion

In this PRISMA-DTA systematic review and diagnostic meta-analysis, ML-based models demonstrated pooled discrimination for postoperative facial nerve outcomes and hearing preservation after VS surgery that was generally higher in training than in held-out testing, with variable performance across studies and limited external validation. These findings are best read as a characterisation of the field’s readiness for synthesis rather than a deployable estimate: data-driven models capture multivariable associations relevant to functional outcomes, but the reliance on the best model per study, the predominance of internal validation, the significant hearing small-study effects, and the outcome-definition heterogeneity together mean a single transportable accuracy figure cannot yet be responsibly reported for either outcome [7, 9].

### What the synthesis does and does not establish

Three signals appear robust: discrimination is reproducibly moderate-to-good (best-model AUC 0.91 facial, 0.92 hearing; all-models test AUC 0.81 facial); the influential predictors converge on a small, biologically coherent set (tumour size, age, tumour location, and baseline hearing status) recurring across cohorts and algorithms; and the gap between apparent and held-out performance, with the significant hearing Deeks asymmetry, marks optimism and selective reporting, not an inability to model the biology, as the dominant failure mode. Against these, heterogeneity in case-mix, outcome definition and follow-up, and validation design constrains synthesis, and because attribution methods differ across studies a quantitative synthesis of feature importance is not yet feasible, itself a finding. Until these axes are standardised, meta-analysis can characterise the field’s readiness, as here, but should not be read as yielding a clinically deployable estimate.

### Mechanistic framing: why facial and cochlear outcomes remain difficult to predict

Functional outcomes after VS resection reflect a complex interaction between tumour biology and anatomy, host vulnerability, and operative metrics. Facial nerve dysfunction may arise from direct mechanical trauma, traction or ischaemia during dissection, thermal injury, or devascularisation with risk magnified by tumour size, adherence, and altered arachnoid planes [33, 34]. Progressive tumour enlargement increases nerve tension and may stretch or “ribbon” the facial nerve along the tumour capsule, particularly at the *porus acusticus* where confinement within the internal auditory canal creates a pressure bottleneck that obscures the dissection corridor [35]. Larger tumours may also compromise nerve microvasculature, either through direct effacement or secondary ischaemia during operative manipulation [33]. Consistent with this anatomical rationale, growth toward the brainstem has emerged as a predictive feature in ML-based models [32]. IONM mitigates some of these risks by providing dynamic feedback, yet it remains an imperfect proxy for delayed neuropraxia and longer-term recovery [36–38].

Hearing outcomes are similarly multifactorial. Preservation depends not only on cochlear nerve continuity but also on cochlear perfusion and microvascular integrity, susceptibility of the inner ear to brief ischaemic intervals, and the cumulative effects of traction, drilling, and cerebellopontine angle manipulation [39]. Tumour size and preoperative hearing metrics likely reflect both tumour-induced cochlear dysfunction and baseline cochlear reserve; when reserve is already limited, even modest perturbation due to nerve manipulation, transient ischemia, or postoperative inflammation may result in disproportionate functional decline. In this regard, age and baseline hearing status are not independent, given the contribution of presbycusis to reduced recovery potential [40]. These mechanisms provide a biological rationale for why purely coarse predictors (such as maximal diameter alone) often underperform at the individual patient level, and why outcome prediction tends to require higher-dimensional representations of tumour-nerve-labyrinth relationships [3, 7, 8].

### Why machine learning can outperform conventional regression without guaranteeing clinical value

A central advantage of ML is its capacity to model nonlinear effects and feature interactions that are likely to be present in skull base surgery (such as the interaction between tumour size, approach, baseline function, and IONM trajectories) [41]. Traditional univariate analyses have generally demonstrated modest associations between individual risk factors and postoperative functional outcomes, with effect sizes insufficient for reliable patient-level prognostication when used in isolation [10–12]. More contemporary multivariable and nomogram-based models have improved discrimination, with reported AUCs approximating 0.80 in selected cohorts [13–15]. In this context, the apparent advantage of ML likely reflects its ability to capture nonlinear interactions and higher-order feature relationships not easily represented by prespecified linear terms [16].

However, this same flexibility increases the risk that apparent performance reflects dataset-specific structure rather than stable biology – particularly when studies rely on internal cross-validation or small held-out test sets [42]. This is especially relevant in VS surgery, where case-mix varies across centers (tumour size distribution, approach preferences, extent-of-resection philosophy, and IONM protocols), and where outcome definitions and follow-up windows are not uniform. In this setting, discrimination can be inflated by internal tuning and will often degrade when models are transported [32].

### Feature representation: what radiomics and automated pipelines may be capturing

Several included studies incorporate radiomic or deep-feature representations derived from preoperative imaging. Mechanistically, these approaches may encode higher-order descriptors of tumour phenotype and local anatomy (heterogeneity, margins, interface complexity, and spatial relationships) that are not captured by routine semantic variables and that plausibly correlate with dissection difficulty, adherence, and nerve vulnerability [26, 29, 31].

That said, radiomics is sensitive to acquisition and preprocessing (scanner parameters, sequences, segmentation, normalisation), which can compromise transportability unless pipelines are standardised and validated across sites. Where external validation is absent or limited, it remains unclear whether reported gains reflect robust signal or center-specific imaging “fingerprints” [32, 42].

### Interpretability and “what the model learned”

Only a subset of studies report explainability or feature-importance methods. In this clinical context, interpretability is not cosmetic: it is necessary to evaluate whether models rely on plausible determinants (such as baseline House-Brackmann grade, tumour size/extension, hearing metrics, approach) versus proxies for practice style or imaging artefacts. Explainable ML approaches for long-term facial nerve outcome prediction represent an important step, but explanation does not substitute for external validation and calibration. Where reported (explainable-AI in Przepiorka et al.; radiomic or impurity-based importance elsewhere), semantic top predictors (tumour size, baseline House–Brackmann grade, hearing metrics, brainstem extension) aligned with established prognostic determinants, whereas radiomic-texture-dominated models rank correlates of uncertain clinical meaning [43–45].

### Clinical translation: aligning prediction with decision points

From a clinical perspective, the relevant question is not whether a model achieves a higher AUC in retrospective testing, but whether it supports decisions that matter: candidacy for surgery versus observation or radiosurgery, approach selection, extent-of-resection strategy counselling regarding functional trade-offs, and postoperative rehabilitation planning. Contemporary management of VS has shifted toward individualised, function-preserving strategies, with observation or stereotactic radiosurgery frequently favored for small, minimally symptomatic tumours and microsurgical resection reserved for larger or progressive lesions [5, 46, 47]. In this context, reliable prediction of facial and hearing outcomes could meaningfully inform treatment selection – particularly when balancing oncologic control against functional preservation. However, such models must demonstrate reproducibility, calibration, and transportability before influencing decision-making. Discrimination alone is insufficient: calibration, decision-curve analysis, and prospective external validation are prerequisites, and calibration was reported in a minority of studies and decision-curve analysis in essentially none [6, 7].

### Limitations of the evidence base

The included literature is dominated by retrospective cohorts with heterogeneity in inclusion criteria, surgical approaches, adjunct monitoring, and outcome assessment. Although facial nerve function was uniformly graded using the House-Brackmann scale, follow-up intervals varied substantially, conflating immediate postoperative weakness, delayed facial palsy, and long-term recovery. These represent biologically distinct phenomena (delayed palsy, for example, may reflect inflammatory edema or viral reactivation) and models trained at a single timepoint may therefore misclassify evolving outcomes. Hearing endpoints were similarly heterogeneous, contributing to inter-model variability.

Most studies relied on internal split validation or cross-validation, with limited external testing, raising concerns for optimism bias and restricted transportability. Reporting of calibration, missing data handling, and prespecified threshold selection was inconsistent, aligning with common methodological weaknesses captured by PROBAST assessments. Small sample sizes and limited external validation constrain inference about transportability, and comparisons across algorithm families are difficult to interpret because performance depends as much on feature representation and validation design as on classifier architecture. As we extracted multiple models per cohort, non-independence may also inflate pooled precision despite our secondary highest-performing-model analysis. Furthermore, by focusing on preoperative prediction, we did not evaluate intraoperative or early postoperative models, which incorporate electrophysiologic thresholds and early facial function and may demonstrate higher apparent discrimination, albeit with different clinical utility [48].

Collectively, these factors limit inference regarding real-world performance and underscore the need for standardised outcome definitions, longitudinal endpoints, and rigorous external validation.

## Conclusions

ML-based models show promising discrimination for predicting facial nerve and hearing outcomes after VS surgery, but current evidence is limited by heterogeneity, inconsistent validation, and incomplete reporting of calibration and decision relevance. Robust clinical adoption will require externally validated, standardised, and interpretable models aligned with real treatment thresholds and the contemporary functional-preservation paradigm in skull base oncology.

## Electronic supplementary material

Below is the link to the electronic supplementary material.

Supplementary Material 1

## Data Availability

No datasets were generated or analysed during the current study.

## Abbreviations

AAO-HNS: American Academy of Otolaryngology-Head and Neck Surgery
AUC: Area Under the Curve
CI: Confidence Interval
DT: Decision Tree
DL: Deep Learning
dOR: Diagnostic Odds Ratio
EM: Ensemble Methods
GLMM: Generalised Linear Mixed Model
IONM: Intraoperative Neurophysiological Monitoring
KNN: K-Nearest Neighbors
LR: Logistic Regression
ML: Machine Learning
MRI: Magnetic Resonance Imaging
MR: Microsurgical Resection
NB: Naïve Bayes
NLR: Negative Likelihood Ratio
NN: Neural Network
PLR: Positive Likelihood Ratio
SROC: Summary Receiver Operating Characteristic
SVM: Support Vector Machine
VS: Vestibular Schwannoma
