# Supplementary material for: Machine learning for functional outcome prediction after vestibular schwannoma surgery: a systematic review and diagnostic test accuracy meta-analysis
Source: J Neurooncol. 2026 Aug 24;179(2):52. doi: 10.1007/s11060-026-05747-5 (PMC13503505; doi:10.1007/s11060-026-05747-5)
Supplement: Supplementary file 1 [file 11060_2026_5747_MOESM1_ESM.docx]

| **Database** | **Search Strategy** |
| --- | --- |
| PubMed  (n=232) | ("vestibular schwannoma" OR "acoustic neuroma" OR "acoustic neurinoma" OR "vestibulocochlear nerve tumor*" OR "VIII nerve schwannoma")  AND  ("machine learning" OR "artificial intelligence" OR AI OR "deep learning" OR "neural network*" OR "convolutional neural network*" OR CNN* OR radiomics OR "radiomic feature*" OR "predictive model*" OR "prediction model*" OR algorithm* OR "classification model*" OR "support vector machine*" OR SVM OR "random forest*" OR "gradient boosting" OR XGBoost)  AND  (predict* OR prognos* OR "outcome prediction" OR "clinical outcome*")  AND  ("hearing preservation" OR hearing OR deafness OR audiometr* OR "facial nerve" OR "facial nerve function" OR "facial palsy" OR "facial paralysis" OR "House-Brackmann") |
| Embase  (n=399) | ("vestibular schwannoma" OR "acoustic neuroma" OR "acoustic neurinoma" OR "vestibulocochlear nerve tumor*" OR "VIII nerve schwannoma" )  AND  ("machine learning" OR "artificial intelligence" OR AI OR "deep learning" OR "neural network*" OR "convolutional neural network*"OR CNN* OR radiomics OR "radiomic feature*" OR "predictive model*" OR "prediction model*" OR algorithm* OR "classification model*" OR "support vector machine*" OR SVM OR "random forest*" OR "gradient boosting" OR XGBoost )  AND  (predict* OR prognos* OR "outcome prediction" OR "clinical outcome*" )  AND  ("hearing preservation" OR hearing OR deafness OR audiometr* OR "facial nerve" OR "facial nerve function" OR "facial palsy" OR "facial paralysis" OR House-Brackmann) |
| CENTRAL  (n=24) | ("vestibular schwannoma" OR "acoustic neuroma" OR "acoustic neurinoma" OR ("vestibulocochlear nerve" NEXT tumor*) OR "VIII nerve schwannoma" )  AND  ("machine learning" OR "artificial intelligence" OR AI OR "deep learning" OR ("neural" NEXT network*) OR ("convolutional neural" NEXT network*) OR CNN* OR radiomics OR ("radiomic" NEXT feature*) OR ("predictive" NEXT model*) OR ("prediction" NEXT model*) OR algorithm* OR ("classification" NEXT model*) OR ("support vector" NEXT machine*) OR SVM OR ("random" NEXT forest*) OR "gradient boosting" OR XGBoost )  AND  (predict* OR prognos* OR "outcome prediction" OR ("clinical" NEXT outcome*) )  AND  ("hearing preservation" OR hearing OR deafness OR audiometr* OR "facial nerve" OR "facial nerve function" OR "facial palsy" OR "facial paralysis" OR House-Brackmann ) |

**Supplementary Table 1*:* Search strategies**

Full electronic search strategies used to identify relevant studies for this review, reported for each database. n = number of articles returned.

| **Family** | **Algorithms** | **Description** |
| --- | --- | --- |
| Regression models (RM) | Logistic regression (LR)  Generalized linear model (GLM) | Parametric models that estimate the relationship between predictors and outcome via a specified link function (such as logit for binary outcomes). Often used as transparent baselines and to quantify adjusted associations. |
| Instance-based methods | K-nearest neighbors (KNN) | Non-parametric learners that classify a case using the labels of the most similar training instances under a chosen distance metric. Performance depends strongly on feature scaling and neighborhood choice (k). |
| Support vector machines (SVMs) | SVM | Margin-based classifiers that learn a separating hyperplane (or kernelized boundary) maximizing class separation. Effective in high-dimensional settings but require careful kernel/regularization tuning. |
| Decision trees (DT) | DT  CART  SMOTE-CART | Recursive partitioning models that split predictors into decision rules to form an interpretable tree. SMOTE-CART denotes a pipeline variant where SMOTE is applied to address class imbalance before fitting a tree |
| Ensemble methods (EM) | Random forest (RF)  Bagging  AdaBoost  Gradient boosting machine (GBM)  Extreme gradient boosting (XGBoost)  Light gradient boosting machine (LightGBM)  SMOTE-XGB  Extra trees (ET) | Combine multiple base learners to improve discrimination and robustness (bagging reduces variance; boosting reduces bias). SMOTE-XGB denotes oversampling for class imbalance followed by boosted trees. |
| Deep learning (DL) | Deep neural network (DNN)  SMOTE-DNN  1DCNN  Artificial neural network (ANN)  Convolutional neural network (CNN) | Multi-layer neural architectures that learn nonlinear feature representations; CNN/1DCNN use convolutional filters to exploit local structure in spatial/temporal signals. SMOTE-DNN denotes an imbalance-handling pipeline variant where SMOTE precedes network training. |
| Naïve Bayes (NB) | NB | Probabilistic classifiers based on Bayes’ rule with a conditional independence assumption between features. |

**Supplementary Table 2: Summary of statistical and machine learning-based approaches**

Summary of statistical and machine learning (ML)-based models employed by studies included in this meta-analysis. Algorithms are grouped into broad “families” based on shared underlying model features. Abbreviations: AdaBoost, adaptive boosting; ANN, artificial neural network; CART, classification and regression tree; CNN, convolutional neural network; DL, deep learning; DT, decision tree; EM, ensemble methods; ET, extremely randomized trees (extra trees); GBM, gradient boosting machine; GLM, generalized linear model; KNN, k-nearest neighbors; LR, logistic regression; LightGBM, light gradient boosting machine; NB, naive Bayes; RM, regression models; RF, random forest; SMOTE, synthetic minority over-sampling technique; SVM, support vector machine; XGBoost, extreme gradient boosting.

**Supplementary Table 3: Risk of bias assessment**

Summary of risk of bias assessment for prediction model studies (PROBAST), reported by individual domain and overall risk of bias.

**Hearing preservation (training data)**

| Sensitivity | 0.83 (95% CI: 0.74 to 0.89) |
| --- | --- |
| Specificity | 0.85 (95% CI: 0.71 to 0.93) |

| Outcome | № of studies (№ of patients) | Study design | Factors that may decrease certainty of evidence | | | | | Effect per 1,000 patients tested | Test accuracy CoE |
| --- | --- | --- | --- | --- | --- | --- | --- | --- | --- |
|  |  |  | Risk of bias | Indirectness | Inconsistency | Imprecision | Publication bias | Pre-test probability 50% |  |
| **True positives** | 4 studies 700 patients | cohort & case-control type studies | serious^a^ | not serious | not serious | not serious | none | 415 (370 to 445) | ⨁⨁◯◯ Low^a^ |
| **False negatives** |  |  |  |  |  |  |  | 85 (55 to 130) |  |
| **True negatives** | 4 studies 670 patients | cohort & case-control type studies | serious^a^ | not serious | not serious | not serious | none | 425 (355 to 465) | ⨁⨁◯◯ Low^a^ |
| **False positives** |  |  |  |  |  |  |  | 75 (35 to 145) |  |

**Facial nerve dysfunction (training data)**

| Sensitivity | 0.82 (95% CI: 0.78 to 0.86) |
| --- | --- |
| Specificity | 0.70 (95% CI: 0.63 to 0.77) |

| Outcome | № of studies (№ of patients) | Study design | Factors that may decrease certainty of evidence | | | | | Effect per 1,000 patients tested | Test accuracy CoE |
| --- | --- | --- | --- | --- | --- | --- | --- | --- | --- |
|  |  |  | Risk of bias | Indirectness | Inconsistency | Imprecision | Publication bias | Pre-test probability 50% |  |
| **True positives** | 3 studies 549 patients | cohort & case-control type studies | serious^b^ | not serious | not serious | not serious | none | 410 (390 to 430) | ⨁⨁⨁◯ Moderate^a^ |
| **False negatives** |  |  |  |  |  |  |  | 90 (70 to 110) |  |
| **True negatives** | 3 studies 1071 patients | cohort & case-control type studies | serious^b^ | not serious | not serious | not serious | none | 350 (315 to 385) | ⨁⨁⨁◯ Moderate^a^ |
| **False positives** |  |  |  |  |  |  |  | 150 (115 to 185) |  |

**Facial nerve dysfunction (test data)**

| Sensitivity | 0.85 (95% CI: 0.75 to 0.91) |
| --- | --- |
| Specificity | 0.83 (95% CI: 0.77 to 0.87) |

| Outcome | № of studies (№ of patients) | Study design | Factors that may decrease certainty of evidence | | | | | Effect per 1,000 patients tested | Test accuracy CoE |
| --- | --- | --- | --- | --- | --- | --- | --- | --- | --- |
|  |  |  | Risk of bias | Indirectness | Inconsistency | Imprecision | Publication bias | Pre-test probability 50% |  |
| **True positives** | 5 studies 635 patients | cohort & case-control type studies | serious^c^ | not serious | not serious | not serious | none | 425 (375 to 455) | ⨁⨁⨁◯ Moderate^a^ |
| **False negatives** |  |  |  |  |  |  |  | 75 (45 to 125) |  |
| **True negatives** | 5 studies 1188 patients | cohort & case-control type studies | serious^c^ | not serious | not serious | not serious | none | 415 (385 to 435) | ⨁⨁⨁◯ Moderate^a^ |
| **False positives** |  |  |  |  |  |  |  | 85 (65 to 115) |  |

**Hearing preservation (best-performing model)**

| Sensitivity | 0.88 (95% CI: 0.83 to 0.92) |
| --- | --- |
| Specificity | 0.96 (95% CI: 0.80 to 0.99) |

| Outcome | № of studies (№ of patients) | Study design | Factors that may decrease certainty of evidence | | | | | Effect per 1,000 patients tested | Test accuracy CoE |
| --- | --- | --- | --- | --- | --- | --- | --- | --- | --- |
|  |  |  | Risk of bias | Indirectness | Inconsistency | Imprecision | Publication bias | Pre-test probability 50% |  |
| **True positives** | 3 studies 164 patients | cohort & case-control type studies | serious^a^ | not serious | not serious | not serious | none | 440 (415 to 460) | ⨁⨁◯◯ Low^a^ |
| **False negatives** |  |  |  |  |  |  |  | 60 (40 to 85) |  |
| **True negatives** | 3 studies 176 patients | cohort & case-control type studies | serious^a^ | not serious | not serious | not serious | none | 480 (400 to 495) | ⨁⨁◯◯ Low^a^ |
| **False positives** |  |  |  |  |  |  |  | 20 (5 to 100) |  |

**Facial nerve dysfunction (best-performing model)**

| Sensitivity | 0.89 (95% CI: 0.81 to 0.94) |
| --- | --- |
| Specificity | 0.86 (95% CI: 0.81 to 0.89) |

| Outcome | № of studies (№ of patients) | Study design | Factors that may decrease certainty of evidence | | | | | Effect per 1,000 patients tested | Test accuracy CoE |
| --- | --- | --- | --- | --- | --- | --- | --- | --- | --- |
|  |  |  | Risk of bias | Indirectness | Inconsistency | Imprecision | Publication bias | Pre-test probability 50% |  |
| **True positives** | 6 studies 311 patients | cohort & case-control type studies | serious^d^ | not serious | not serious | not serious | none | 445 (405 to 470) | ⨁⨁⨁◯ Moderate^a^ |
| **False negatives** |  |  |  |  |  |  |  | 55 (30 to 95) |  |
| **True negatives** | 6 studies 647 patients | cohort & case-control type studies | serious^d^ | not serious | not serious | not serious | none | 430 (405 to 445) | ⨁⨁⨁◯ Moderate^a^ |
| **False positives** |  |  |  |  |  |  |  | 70 (55 to 95) |  |

Explanations

a. All 'unclear' ROB on PROBAST

b. 1 ‘high’ + 2 ‘unclear’ ROB on PROBAST

c. 4 ‘unclear’ + 1 ‘low’ ROB on PROBAST

d. 1 ‘high’ + 4 ‘unclear’ + 1 ‘low’ ROB on PROBAST

**Supplementary Table 4: Certainty of evidence (GRADE)**

GRADE assessment of certainty of evidence for each significant outcome in the present review.

| **Study** | **Follow-up / outcome timepoint** | **Side (R/L)** | **Preoperative Koos grade (I/II/III/IV)** | **Preoperative HB grade (I/II/III/IV/V/VI)** | **Postoperative HB grade, long-term (I/II/III/IV/V/VI)** | **Postoperative PTA (dB)** | **Postoperative WRS (%)** |
| --- | --- | --- | --- | --- | --- | --- | --- |
| Song (2025) | Short-term | NR | NR | NR | NR | NR | NR |
| Przepiorka (2025) | Long-term (≥12 months) | 129/127 | NR | 209/38/5/1/0/3 | 80/90/33/16/11/26 | NR | NR |
| Heman-Ackah (2024) | 6 months | 114/128 | NR | NR | I: 206; II-VI: 36 | NR | NR |
| Zohdy (2024) | Short- & long-term | 35/36 | 11/22/18/20 | NR | 33/14/14/0/0/0 | NR | NR |
| Wang M-Y (2023) | Short-term (Day 7) | 52/58 | NR | 62/9/0/0/0/0 | NR | NR | NR |
| Wang J (2022) | NR | NR | NR | NR | NR | NR | NR |
| Yang (2023) | NR | NR | NR | NR | NR | NR | NR |
| Dixon (2022) | NR | NR | 84/59/1 | NR | NR | NR | NR |
| Cha (2020) | 6 months | 29/21 | NR | NR | NR | 62.5 ± 41.7 | 50.1 ± 42.3 |
| Lim (2024) | 6 months | 38/35 | NR | NR | NR | NR | NR |

**Supplementary Table 5: Expanded cohort characteristics.**

Abbreviations: R, right; L, left; HB, House–Brackmann; PTA, pure tone audiometry; WRS, word recognition score; NR, not reported.

| **Study** | **Radiomics pipeline** | **Imaging modality** | **Segmentation method** | **Feature-extraction software** |
| --- | --- | --- | --- | --- |
| Song (2025) | Manual | MRI | Automatic 2.5D Trans-UNet segmentation with manual correction (3D Slicer) | Pyradiomics |
| Przepiorka (2025) | Automated & manual | MRI | NR | NR |
| Heman-Ackah (2024) | Manual | MRI | Manual linear measurements (no volumetric segmentation) | NR |
| Zohdy (2024) | Manual | MRI | NR | NR |
| Wang M-Y (2023) | Manual | MRI | Manual volumetric segmentation | Pyradiomics |
| Wang J (2022) | Manual | MRI | NR | NR |
| Yang (2023) | NR | MRI | NR | NR |
| Dixon (2022) | Manual | MRI | NR | NR |
| Cha (2020) | NR | MRI | NR | NR |
| Lim (2024) | Manual | MRI | Manual volumetric segmentation (MRIcro) | Pyradiomics |

**Supplementary Table 6: Radiomics and imaging methodology.**

Abbreviations: MRI, magnetic resonance imaging; GLDM/GLRLM/GLSZM, grey-level dependence/run-length/size-zone matrices; NR, not reported.

**Supplementary Figure 1: Predictive performance for hearing preservation (best performing model)**

Forest plots (random effects model) reporting (A) sensitivity, (B) specificity, and (C) diagnostic odds ratio (DOR) for prediction of hearing preservation post-surgery, for best-performing model. Individual study estimates are shown as squares proportional to study weight. Horizontal lines indicate 95% confidence intervals. Diamond represents pooled estimate for overall effect. (D) Summary receiver operating characteristic (SROC) curve showing the overall predictive performance for HP, with the summary operating point (pooled sensitivity and specificity), 95% confidence region, and the area under the curve (AUC). IV = inverse variance; GLMM = generalized linear mixed model.

**Supplementary Figure 2: Predictive performance for facial nerve dysfunction (best performing model)**

Forest plots (random effects model) reporting (A) sensitivity, (B) specificity, and (C) diagnostic odds ratio (DOR) for prediction of FND post-surgery, for best-performing model. Individual study estimates are shown as squares proportional to study weight. Horizontal lines indicate 95% confidence intervals. Diamond represents pooled estimate for overall effect. (D) Summary receiver operating characteristic (SROC) curve showing the overall predictive performance for FND, with the summary operating point (pooled sensitivity and specificity), 95% confidence region, and the area under the curve (AUC). IV = inverse variance; GLMM = generalized linear mixed model.

**Supplementary Figure 3: Fagan nomograms**

Fagan plots demonstrating the change from pre-test to post-test probability of (A) hearing preservation and (B) facial nerve dysfunction using pooled likelihood ratios. Solid red lines indicate the post-test probability following a positive test result, while dashed blue lines indicate the post-test probability following a negative test result.

**Supplementary Figure 4: Publication bias assessment**

Deeks’ funnel plots for assessment of publication bias for (A) hearing outcomes and (B) facial nerve outcomes. Diagnostic odds ratio is plotted against the inverse square root of the effective sample size. Circles represent individual studies, and the dashed line denotes the weighted linear regression line used in Deeks’ asymmetry test.
